# RETRACTED: Fusco et al. Formyl Peptide Receptor 1 Signaling in Acute Inflammation and Neural Differentiation Induced by Traumatic Brain Injury. *Biology* 2020, *9*, 238

**DOI:** 10.3390/biology14121656

**Published:** 2025-11-24

**Authors:** Roberta Fusco, Enrico Gugliandolo, Rosalba Siracusa, Maria Scuto, Marika Cordaro, Ramona D’Amico, Maurizio Evangelista, Angelo Peli, Alessio Filippo Peritore, Daniela Impellizzeri, Rosalia Crupi, Salvatore Cuzzocrea, Rosanna Di Paola

**Affiliations:** 1 Department of Chemical, Biological, Pharmaceutical and Environmental Sciences, University of Messina, Viale Ferdinando Stagno D’Alcontres, n 31, 98166 Messina, Italy; rfusco@unime.it (R.F.); egugliandolo@unime.it (E.G.); rsiracusa@unime.it (R.S.); cordarom@unime.it (M.C.); rdamico@unime.it (R.D.); aperitore@unime.it (A.F.P.); dimpellizzeri@unime.it (D.I.); rcrupi@unime.it (R.C.); 2 Department of Biomedical and Biotechnological Sciences, University of Catania, Piazza Università, n 2, 95131 Catania, Italy; mary-amir@hotmail.it; 3 Institute of Anaesthesiology and Reanimation, Catholic University of the Sacred Heart, A Gemelli Hospital, 00168 Roma, Italy; maurizio.evangelista@unicatt.it; 4 Department of Veterinary Medical Sciences, Alma Mater Studiorum, University of Bologna, Via Zamboni n 33, 40126 Bologna, Italy; angelo.peli@unibo.it; 5 Department of Pharmacological and Physiological Science, Saint Louis University School of Medicine, 1402 South Grand Blvd, St. Louis, MO 63104, USA

The journal retracts the article, “Formyl Peptide Receptor 1 Signaling in Acute Inflammation and Neural Differentiation Induced by Traumatic Brain Injury” [1], cited above.

Following publication, concerns were brought to the attention of the Editorial Office regarding the integrity of the images presented in this publication [1].

Adhering to our complaints procedure, an investigation was conducted by the Editorial Office and Editorial Board that confirmed an overlap between Figures 2B and 2C, and between Figures 6A and 6B, representing different experimental groups published in [1]. Additional concerns related to inconsistencies between the reported sample size and the sample size included in the original data were identified by the Editorial Board. While the authors fully cooperated during the investigation, the concerns raised could not be adequately resolved. Consequently, the Editorial Board has lost confidence in the rigor of the manuscript and decided to retract this publication [1], as per MDPI’s retraction policy (https://www.mdpi.com/ethics#_bookmark30 (accessed on 19 November 2025)).

This retraction was approved by the Editor-in-Chief of the journal *Biology*.

The authors did not agree to this retraction.
